# Management Challenges of Life-Threatening Thrombosis and Hemorrhage in a Jehovah’s Witness Patient With Hereditary Hemorrhagic Telangiectasia: A Case Report

**DOI:** 10.7759/cureus.98904

**Published:** 2025-12-10

**Authors:** Ahmed Naeem, Sughra M Mangrio, Aaiza Azhar, Usmaan Khan

**Affiliations:** 1 General Internal Medicine, Russells Hall Hospital, Dudley, GBR; 2 Internal Medicine, Independent Research, Dudley, GBR; 3 Internal Medicine, CMH Lahore Medical College and Institute of Dentistry, Lahore, PAK

**Keywords:** complex anticoagulation management, hereditary hemorrhagic telangiectasia (hht), inherited bleeding diathesis, jehovah's witness, pulmonary embolism

## Abstract

Hereditary hemorrhagic telangiectasia (HHT) is a rare autosomal dominant vascular disorder characterized by mucocutaneous telangiectasias and arteriovenous malformations (AVMs) affecting multiple organ systems. Clinical manifestations commonly include recurrent epistaxis and chronic iron deficiency anemia, while complications such as gastrointestinal (GI) bleeding, high-output cardiac failure, stroke, and venous thromboembolism may occur. Management can be further complicated when an anticoagulant is indicated. We present a case of an 84-year-old female Jehovah’s Witness with known HHT, heart failure, and atrial fibrillation who was admitted with worsening exertional dyspnea. Baseline investigations revealed type 1 respiratory failure and elevated D-dimer, and imaging confirmed the diagnosis of massive pulmonary embolism with right heart strain. In light of this diagnosis, she was started on split-dose low molecular weight heparin (LMWH); however, management was significantly constrained by her history of recurrent epistaxis and new-onset GI bleeding, as well as refusal of blood transfusion in accordance with her religious beliefs. Despite standard interventions by ENT to control her nasal bleeding, including cauterization and topical medications, she developed melena and continued to regress afterwards. Anticoagulation was hence discontinued at that time to minimize any further risk of bleeding, but her condition continued to deteriorate. Blood transfusion was discussed but was declined, consistent with the patient’s advance directive. Supportive and palliative measures were initiated, but the patient subsequently passed away following clinical decline. This case highlights the complex clinical and ethical challenges of balancing thrombosis and hemorrhage in patients with HHT, particularly when standard interventions are not an option. It highlights the importance of individualized risk-benefit assessment, multidisciplinary involvement, and early engagement with hospital liaison services in Jehovah’s Witness patients to make sure that treatment aligns with patients' personal beliefs, and this can prevent any delays in considering life-saving treatment, making sure that patients are well informed of the possibility of any adverse outcome beforehand. Additionally, it reflects the need for continued research into safe and effective alternatives to transfusion of blood products in patients where current standard treatment options, although effective, do not have the same clinical impact.

## Introduction

Hereditary hemorrhagic telangiectasia (HHT), also known as Osler-Weber-Rendu disease, is an autosomal dominant genetic disorder, which means that, if a parent has HHT, there is a 50% chance that each child might inherit the condition, regardless of the gender of the parent or the child. It has a prevalence of approximately one in 9,400 individuals, which varies across different factors such as age, gender, socioeconomic status, and geographical location, as per a study conducted in 2010 in the United Kingdom [1].

It is characterized by certain clinical features, including formation of arterio-venous malformations (AVMs) in solid organs such as lungs, liver, brain, gastrointestinal (GI) tract, and, rarely, in the spine, kidney, and pancreas [2]. These often lead to complications such as stroke, high-output cardiac failure, and acute or chronic GI bleed leading to iron deficiency anemia, and, in case of spinal involvement, might lead to paralysis. These AVMs might also manifest as smaller mucocutaneous telangiectasias, mostly a few millimeters in diameter, such as small pin-point lesions, which blanch on pressure, but they lead to frequent and brisk bleeding even on slight trauma. Telangiectasias are focal dilatations of postcapillary venules, which dilate and extend through the dermis with excessive layers of smooth muscle without elastic fibers. They lack capillaries and are directly connected to arterioles. They can be seen most notably in the nasal mucosa, leading to recurrent episodes of epistaxis, or on the lips, tongue, fingers, and face. AVMs are quite similar but are direct connections of veins to arteries and hence are much larger in size. These vessels, due to their abnormal structure and high perfusion pressure, are more prone to bleeding due to multiple factors, including direct connection of the venule with the artery and thinning of the vessel walls.

The pathogenesis includes mutations in specific genes involved in the proper development of blood vessels, including endoglin (ENG), activin receptor-like kinase type 1 (ACVRL1), or SMAD4 genes, leading to HHT 1, HHT 2, and juvenile polyposis HHT, respectively. In most cases, individuals remain asymptomatic in early life, with a majority showing at least some clinical signs and symptoms later in life, at around the age of 30 or 40 [3]. Diagnosis is usually based on the presence of certain features falling on specific criteria called the Curacao criteria, which entail four indicators: spontaneous and recurrent nosebleeds, mucocutaneous telangiectasis, visceral AVMs, and a family history of HHT in a first-degree relative. A score of three or four suggests a "definite" diagnosis, while two indicates a "possible" diagnosis. It can also be confirmed via molecular genetic testing, which is particularly helpful in patients with a "possible" clinical diagnosis of HHT [4]. The criteria had a sensitivity of 68% and specificity of 98%, with reduced sensitivity in younger individuals, which increases with age.

Treatment usually entails supportive measures. For epistaxis, the initial approach includes topical moisturizing therapies and hemostatic products; if these fail, then antifibrinolytic therapy (tranexamic acid) and ablation therapy are considered. If all of the above remain unsuccessful, then the last resort is systemic anti-angiogenic agents such as intravenous (IV) bevacizumab, which works by primarily targeting vascular endothelial growth factor (VEGF), reducing the formation of new blood vessels, reducing the incidence of anemia, epistaxis, and high output cardiac failure. These agents are used in addition to and sometimes in place of standard management [5]. Meanwhile, for anemia, iron supplementation (either oral or IV, if oral not tolerated) is first line. Transfusion of red blood cells is considered in those with significant hemodynamic instability or those who are unable to maintain hemoglobin levels despite frequent IV iron transfusion. The mean life expectancy of people with HHT was found to be around 77 years [6], with a high mortality rate as a result of associated complications.

## Case presentation

An 84-year-old female presented to the Accident and Emergency Department of Russells Hall Hospital, Dudley, England, with the complaint of worsening shortness of breath for the past two weeks. She had no associated fever, cough, chest pain, or palpitation. On examination, she was conscious and alert, fine crackles were present on the bases of both lungs, and a characteristic pansystolic murmur was audible all over the precordium but loudest over the tricuspid region with radiation to the axilla. She had bilateral mild non-pitting pedal edema up to her mid-thighs. Jugular venous pressure (JVP) was visible at the mid-neck level. She had an oxygen saturation of 80% on room air and a National Early Warning Score (NEWS) of 2 [7]. She was admitted under the care of an acute medical team and was started on supportive treatment and empirical IV antibiotics to safeguard against any possibility of infection till other differentials were ruled out.

She was a known case of heart failure with preserved ejection fraction (HFpEF) with left ventricular ejection fraction (LVEF) of 45-50% in an echocardiogram done a year ago. Her regular medications included an angiotensin-converting enzyme (ACE) inhibitor (ramipril), statin (atorvastatin), and diuretic (furosemide). Other comorbidities include hypertension, atrial fibrillation (AF), and HHT. In lieu of a CHA2Ds2-VASc score of 4 [8], the patient was previously on a direct oral anticoagulant (DOAC) (i.e., rivaroxaban), but this was discontinued in view of the recurrent episodes of epistaxis, which she struggled with for the last 50 years as a result of HHT and subsequent anemia. Her ORBIT score at the time was around 3, which showed her to be at a moderate risk of bleeding. Other treatment options, including a reduced dose of rivaroxaban or alternative medication such as apixaban, were also considered. Apixaban has shown to be associated with a relatively lower risk of bleeding in patients with HHT, but due to her reluctance to continue anticoagulation, it was discontinued; however, she was advised regarding the potential risk of thromboembolism. Her episodes of epistaxis were mostly managed by first aid measures at home, topical nasal creams, and douches. She was also taking regular oral iron supplements. She was under regular review by the ENT team and the cardiac specialist regarding the above-mentioned issues. As for her lifestyle, the patient was mobile and independent in her daily activities. She was a non-smoker and used to drink alcohol occasionally. Her family history was positive for HHT in first-degree relatives. The patient was diagnosed on the basis of clinical criteria with the presence of three factors: recurrent epistaxis, telangiectasias, and a family history of HHT.

**Figure 1:**
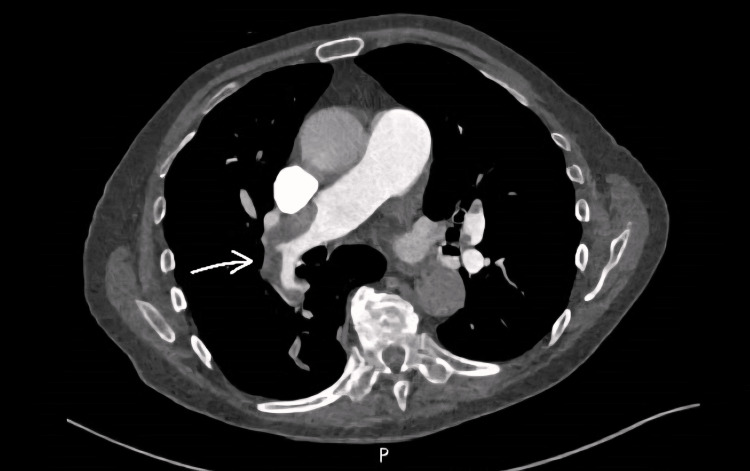
Computed tomography pulmonary angiogram (CTPA) Pulmonary embolism noted involving the right main pulmonary artery (arrow), the right interlobar artery, and multiple segmental arteries on both sides. Diagnosis of massive pulmonary embolism was based on imaging evidence.

**Figure 2:**
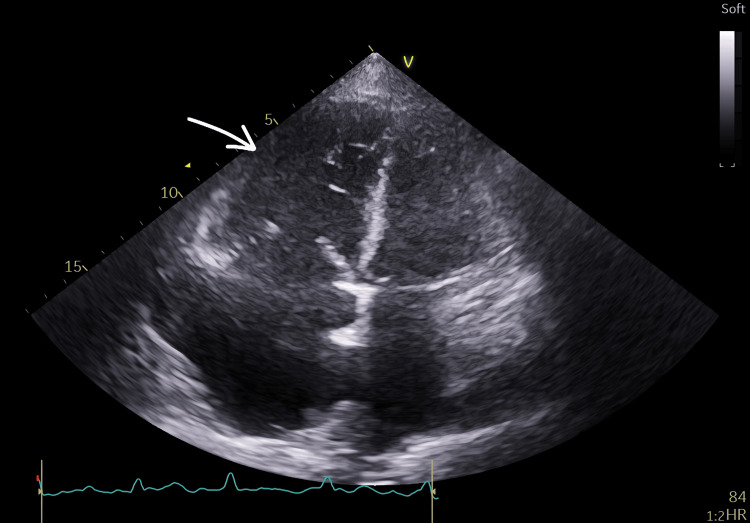
Echocardiogram Echocardiogram report included findings of severely dilated right ventricle (arrow) at the basal and mid-level with preserved basal contractility. Regarding normal left ventricle wall thickness, there was evidence of inter-ventricular septal flattening seen throughout the cardiac cycle, indicating increased right-sided pressure and volume overload. Thickened tricuspid valve with loss of leaflet apposition and severe tricuspid regirgitation noted, and dilated inferior vena cava and left atrium, all suggestive of pulmonary hypertension and right heart strain.

Baseline investigations, as can be seen in Table 1, were reviewed and compared with results from previous months, showing a progressive decline in hemoglobin (which was 100 g/L one month before presentation). Iron studies done two months prior to admission showed chronic iron deficiency anemia. Arterial blood gas analysis demonstrated type one respiratory failure. D-dimer was also markedly elevated at 5,706 ng/mL (reference range: <500 ng/mL). The clotting profile was relatively normal throughout the admission, apart from elevated PT levels. Computed tomography pulmonary angiogram (CTPA) (Figure 1) together with echocardiography (Figure 2) confirmed the diagnosis of a massive pulmonary embolism based on imaging severity, identifying a large obstructive clot in the main pulmonary artery and signs of significant strain on the right heart.

**Table 1:** Laboratory results Hb: hemoglobin, WBC: white blood cells, MCV: mean corpuscular volume, MCH: mean corpuscular hemoglobin, RBC: red blood cells, eGFR: estimated glomerular filtration rate, PT: prothrombin time, INR: international normalized ratio, APTT: activated partial thromboplastin time Laboratory results show a steady decline in hemoglobin levels and fluctuating blood indices throughout admission. The clotting profile was normal apart from raised PT levels. eGFR levels were also below optimal levels. All these variables were consistently monitored during the patient's admission to make any necessary adjustments to treatment.

Test	Reference Range	Unit	Day of presentation	Day 2	Day 4	Day 5	Day 6	Day 9	Day 10
HB	115-165	g/L	89	93	82	85	82	70	58
WBC	3.6-11.0	x 10^9^/L	9.4	8.9	5.9	6.9	6	6.6	10.1
Platelets	140-400	x 10^9^/L	434	475	409	505	442	367	408
MCV	80-100	fL	83.3	82.4	80.5	81.6	83.1	82.9	81.3
MCH	27-32	pg	24.7	24.7	24.3	24.1	23.7	23.5	24.1
RBC	3.80-5.80	x 10^12^/L	3.6	3.76	3.38	3.53	3.5	2.97	2.41
eGFR	>90	mL/min	42	44	44	52	60	60	57
PT	9.5-13.5	seconds	14.0	14.5	13.6	12.6	13.5	-	-
INR	0.8-1.2	ratio	1.2	1.3	1.2	1.1	1.2	-	-
APTT	25-35	seconds	27.6	30.5	30.6	30.8	31.5	-	-
Clauss fibrinogen	1.5-4.5	g/L	2.58	3.17	2.96	3.04	3.12	-	-

Based on the above findings, she was reviewed by the hematology team, and a decision was made to start her on a split dose of low molecular weight heparin (LMWH), enoxaparin 60 mg twice daily, dosed by body weight 1 mg/kg. This split dosing was started keeping in view local trust guidelines regarding dosing in patients of massive pulmonary embolism requiring admission and to potentially minimize any risk of bleeding and improve tolerability in the context of HHT. Alternative strategies, including IVC filters, were not actively considered, since treatment with anticoagulants was a viable option in her case, given that she had no absolute contraindication against anticoagulation, including any significant GI or intracranial bleed. Even with a filter in place, anticoagulation is recommended as soon as possible, as the filter itself does not address the underlying hypercoagulable state and can promote deep vein thrombosis (DVT) formation over time. The patient was counselled regarding the bleeding risk, and she consented to the anticoagulation treatment. In the event of significant bleeding as a consequence of this treatment, the option of transfusion of blood or blood products as a possible treatment option was discussed with the patient and her family. Since the patient was a Jehovah’s Witness, she did not want any form of human blood products (including the four main components of blood: red cells, white cells, platelets, and plasma); however, she was willing to accept prothrombin complex concentrate (PCC) or other alternatives, such as non-blood volume expanders. In the event of bleeding, we were advised to contact the hospital liaison committee for Jehovah’s Witnesses. In light of this and her low hemoglobin levels, she was continued on oral iron replacement therapy (ferrous fumarate 210 mg), and it was decided to optimize treatment with non-blood volume expanders.

The patient experienced mild-to-moderate epistaxis in the days following her admission and starting anticoagulant therapy. She was hence reviewed by the ENT team, anterior rhinoscopy was done, and mild oozing of blood was noted from the right nostril with an active bleeding point. After minimal cleaning with saline, the area was cauterized with silver nitrate, and naseptin (chlorhexidine and neomycin sulfate)-soaked gauze was applied afterwards. No cauterization was performed on the left due to the risk of septal perforation, and it was recommended that the other side would likely need cautery, but this was only to be attempted after a gap of six weeks to avoid the risk of septal perforation.

The patient remained relatively stable over the next few days, with improvement in breathlessness. She was maintaining oxygen saturation above 94%. She had another mild episode of epistaxis later, which resolved on its own, and no major bleed was noted from anywhere else. Examination showed a significant dried clot over the right nasal septum, with no indication for further cautery at the time. Her hemoglobin levels dropped to 70, and it was decided to start her on IV iron infusion (Monofer 1,500 mg/week as per body weight of patient in accordance with trust guidelines) to de-escalate any exacerbation of symptoms due to anemia.

On the 10th day of admission, the patient had two episodes of severe melena, after which she started feeling nauseous and dizzy. She was reviewed and was found to be alert but struggling to retain and repeat information and was slightly confused. Her NEWS score at the time had risen to 5, and repeat hemoglobin levels indicated a significant drop to 58. Keeping this recent episode in view, it was decided to discontinue enoxaparin till further notice until we could assess her condition better. Meanwhile, hematology was consulted regarding reduced dosing options with risk-to-benefit assessment. We contacted both the hospital liaison committee for Jehovah’s Witnesses and the patients’ next of kin, explaining that she would require a blood transfusion to prevent her condition from deteriorating further; however, keeping her religious beliefs in view and advance directive to refuse blood transfusion, we respected her wishes, and she was kept on supportive treatment such as non-blood volume expanders and iron transfusion. A gastroenterology consult was done regarding melena, and whether any further intervention, including upper GI endoscopy, should be considered to establish the site of bleed. However, keeping in view the patient's deteriorating condition, it was decided not pursue it.

The patient’s condition further worsened the following day, with a significant drop in her Glasgow coma scale (GCS), and her NEWS score increased to 10 [9]. Keeping in view her current condition, her prognosis as per the Gold Standards Framework (GSF) prognostic indicator guide [10], and limitations of treatment, after considerable input from the hospital liaison committee, it was decided to start her on palliative care, and her regular medications were optimized. Other treatment options, including antiangiogenic agents, were not considered due to her prognosis and the futility of such options in her case at that point. Unfortunately, on the 12th day of admission, the patient passed away after a progressive clinical decline.

## Discussion

HHT is a rare but severely underdiagnosed genetic disorder with an autosomal dominant pattern of inheritance. The diagnosed frequency is significantly higher in the female population as compared to the male population; this might be reflective of an underdiagnosis due to a lower number of male patients opting to consult with primary care services [1]. It is characterized by vascular dysplasia in multiple organs, leading to excessive bleeding. Characteristic features include AVMs and mucocutaneous telangiectasias. The first and foremost symptom of HHT is usually recurrent epistaxis, mostly presenting around childhood and adolescence [11], leading to complications such as chronic anemia. While HHT is inherently a bleeding disorder, patients are at a higher risk of venous thromboembolism (VTE) and stroke, which is particularly due to high levels of factor VIII and von Willebrand factor (vWF). Furthermore, chronic bleeding predisposes patients to iron deficiency anemia, which independently leads to increased levels of factor VIII, contributing even more to the overall prothrombotic state. The co-existence of high bleeding risk due to fragile blood vessels and increased risk of thrombosis creates a complex clinical dilemma, the management of which requires a careful balance.

In our case, the patient’s management presented itself to be challenging on many fronts. The patient was a diagnosed case of HFpEF, AF, and HHT under the care of multiple specialties, including cardiology, ENT, and gastroenterology. She had a CHA2Ds2-VASc score of 4, which clinically warrants the use of warfarin or DOAC [12], as this is associated with a significantly high risk of stroke and other thrombo-embolic events (approximately 40% risk of developing thromboembolism in the next 10 years [13]). The benefit of preventing cerebrovascular complications generally outweighs the risk of bleeding. Our patient at the time of presentation was on no such therapy. She was previously on rivaroxaban for a while, but due to her recurrent episodes of epistaxis, which were quite bothersome, requiring multiple ENT visits and unsuccessful attempts at cauterization, it was decided initially to reduce the dose of rivaroxaban. Eventually, anticoagulants were discontinued altogether due to the patient’s reluctance to continue treatment.

Heart failure and AF often co-exist, leading to significantly high rates of mortality and morbidity. AF often worsens the symptoms of heart failure over time. Management usually involves anticoagulation and rate control, with significant improvement seen from eventual catheter ablation therapies, which lead to improved survival rates and an overall better quality of life [14,15]. Pulmonary embolism and AF are found to have an almost reciprocal relation, with one leading to the other. In patients with AF not on any proper anti-coagulation therapies, the risk of thromboembolism is almost unavoidable, and an expected complication, such as the formation of a clot in the right atrium, might eventually travel to the pulmonary venous system. Similarly, in pulmonary embolism, due to increased pressure in the pulmonary system, it might lead to pressure overload in the right atrium and eventual arrhythmia [16].

In an acute clinical setting, treatment of pulmonary embolism as per guidelines is with thrombolytics or, if possible, with invasive embolectomy procedures. In our case, the patient was given LMWH (enoxaparin) because thrombolysis was an absolute contraindication given the known bleeding diathesis. Split dosing was utilised rather than once daily dosing. This dosing was considered to be better suited, keeping in mind her previous episodes of nasal bleeds, and certain guidelines also suggest a dosing of 1 mg/kg twice daily for patients diagnosed with massive embolism requiring admission. Additionally, in recent studies, this has shown to be more effective due to better bioavailability, but no significant difference in efficacy or safety has been found, as indicated in a meta-analysis by Niu et al. [17]. This treatment was eventually discontinued during her hospital stay when her condition suddenly deteriorated after multiple episodes of melena, which caused a significant drop in her hemoglobin levels. DOACs have almost always posed as a conundrum when weighing the risk versus benefit of this therapy in patients with HHT. Studies show that patients with HHT with AF or venous thromboembolism on DOAC saw a 75% increase in the frequency of nose bleeds, leading to higher dropout rates and discontinuation from treatment [18].

Studies show that a vast majority of HHT patients succumb to their illness as a result of complications. A cohort study done between 2010 and 2018 by Thompson et al. [19] showed that GI bleeding and anemia were associated with increased mortality, with approximate percentages between 48% and 77%, respectively.

Treatment protocol for anemia mostly follows a step-ladder approach, keeping in view the severity, but mostly requires initial treatment with oral or IV iron supplements, followed by subcutaneous erythropoietin. As a last resort, transfusion of blood products is done. A similar approach was adopted in our case, but due to treatment constraints faced as a result of the patient’s religious beliefs as a Jehovah’s Witness, it was more challenging for us to find avenues to clinically compensate and treat the patient in an optimal manner. There have been case reports where Jehovah’s Witnesses with severe anemia were managed successfully with alternative management strategies such as erythropoietin injections or advanced treatment such as anti-angiogenic medications [20]. Anti-angiogenic agents have been well researched and have proven to be a useful part of "bloodless medicine" practice for patients of HHT in whom bleeding is refractory to other medical options. Specific scenarios where this treatment option can be initiated include the following: failure of topical, local, or ablative procedures; chronic iron deficiency anemia requiring regular transfusion; widespread bleeding sites; and high-output cardiac failure. In our case, one of the limitations that we see was the lack of consideration of this treatment modality earlier on in her case, when standard therapy with blood products was already refused by the patient.

Although treatment with non-blood volume expanders and iron supplementation was used as a supportive measure, once the patient's condition deteriorated, other methods, such as erythropoietin injections or PCC, were not pursued further, as her prognosis at that time was quite poor, and any further aggressive treatment was not deemed beneficial for the patient at that time. The patient’s earlier refusal of blood products, grounded in her Jehovah’s Witness beliefs, was honored after confirming full decision-making capacity and ensuring full understanding of the life-threatening risks. The patient’s autonomy was ethically prioritized, and care was directed towards all permissible bloodless strategies to uphold beneficence and nonmaleficence while respecting her values. Keeping the patient's best interest in mind, it was decided to start her on palliative treatment. Some clinicians may think transfusion to be an absolute necessity for treatment of severe anemia, and refusal of this is perceived as rejection of treatment. It is often difficult to gain consent from patients or relatives in these situations, and in these circumstances, consideration is required not only from a medical point of view but also from an ethical standpoint.

## Conclusions

This case illustrates the complex balance between managing life-threatening thrombosis and recurrent hemorrhage in patients with HHT, particularly when a standard treatment option, such as blood transfusion, is refused for religious reasons. This poses an intricate challenge for us as clinicians and compels us to look beyond the scope of mainstream treatment and consider information at our disposal to make decisions that cater to the patient's wishes. In cases like these, early involvement of the hospital liaison committee for Jehovah's Witnesses should be emphasized to ensure patient autonomy so that alternative options for bloodless medicine can be incorporated in a timely manner. This case underscores several lessons that may be generalized to similar patients, in particular, the importance of exploring alternative treatment options, including IVC filters, early on in patients with HHT and AF in whom the risk of thromboembolism is increased and conventional anticoagulation therapies are not ideal due to risk of recurrent bleeding. Anti-angiogenic agents should also be studied in terms of their overall efficacy and benefit in the prognosis of patients with HHT and severe anemia, particularly those who are not candidates for treatment with blood transfusion. Individualized clinical decision-making and early multidisciplinary involvement are essential to navigate these challenges and optimise treatment. Similarly, clear communication regarding patient values is needed to provide care that is both medically appropriate and ethically respectful.

## References

[1] Donaldson JW, McKeever TM, Hall IP, Hubbard RB, Fogarty AW (2014). The UK prevalence of hereditary haemorrhagic telangiectasia and its association with sex, socioeconomic status and region of residence: a population-based study. Thorax.

[2] McDonald J, Stevenson DA (2021). Hereditary hemorrhagic telangiectasia. GeneReviews.

[3] (2025). Hereditary haemorrhagic telangiectasia. https://dermnetnz.org/topics/hereditary-haemorrhagic-telangiectasia.

[4] McDonald J, Bayrak-Toydemir P, DeMille D, Wooderchak-Donahue W, Whitehead K (2020). Curaçao diagnostic criteria for hereditary hemorrhagic telangiectasia is highly predictive of a pathogenic variant in ENG or ACVRL1 (HHT1 and HHT2). Genet Med.

[5] Iyer VN, Apala DR, Pannu BS (2018). Intravenous bevacizumab for refractory hereditary hemorrhagic telangiectasia-related epistaxis and gastrointestinal bleeding. Mayo Clin Proc.

[6] Donaldson JW, McKeever TM, Hall IP, Hubbard RB, Fogarty AW (2015). Complications and mortality in hereditary hemorrhagic telangiectasia: a population-based study. Neurology.

[7] (2025). National early warning score (NEWS). https://www.mdcalc.com/calc/1873/national-early-warning-score-news.

[8] (2025). CHA₂DS₂-VASc score for atrial fibrillation stroke risk. https://www.mdcalc.com/calc/801/cha2ds2-vasc-score-atrial-fibrillation-stroke-risk.

[9] Jain S, Margetis K, Iverson LM (2025). Glasgow coma scale. StatPearls.

[10] (2025). The Gold Standards Framework: proactive identification guidance (PIG). https://www.goldstandardsframework.org.uk/wp-content/uploads/2025/07/Proactive-Identification-Guidance.pdf.

[11] Berg J, Porteous M, Reinhardt D (2003). Hereditary haemorrhagic telangiectasia: a questionnaire based study to delineate the different phenotypes caused by endoglin and ALK1 mutations. J Med Genet.

[12] Lane DA, Lip GY (2012). Use of the CHA2DS2-VASc and HAS-BLED scores to aid decision making for thromboprophylaxis in nonvalvular atrial fibrillation. Circulation.

[13] Olesen JB, Lip GY, Hansen ML (2011). Validation of risk stratification schemes for predicting stroke and thromboembolism in patients with atrial fibrillation: nationwide cohort study. BMJ.

[14] Darby AE (2014). Management of atrial fibrillation in patients with heart failure. J Atr Fibrillation.

[15] Shiga T (2013). [Atrial fibrillation in patients with heart failure: treatment and management]. Nihon Rinsho.

[16] Bikdeli B, Abou Ziki MD, Lip GY (2017). Pulmonary embolism and atrial fibrillation: two sides of the same coin? A systematic review. Semin Thromb Hemost.

[17] Niu J, Song Y, Li C, Ren H, Zhang W (2020). Once-daily vs. twice-daily dosing of enoxaparin for the management of venous thromboembolism: a systematic review and meta-analysis. Exp Ther Med.

[18] Shovlin CL, Millar CM, Droege F (2019). Safety of direct oral anticoagulants in patients with hereditary hemorrhagic telangiectasia. Orphanet J Rare Dis.

[19] Thompson KP, Nelson J, Kim H (2021). Predictors of mortality in patients with hereditary hemorrhagic telangiectasia. Orphanet J Rare Dis.

[20] Jung J, Lee M, Kang Y, Cho SH (2022). Patient blood management when blood is not an option: a report of two cases. Ann Palliat Med.

